# Revealing the Molecular Portrait of Triple Negative Breast Tumors in an Understudied Population through Omics Analysis of Formalin-Fixed and Paraffin-Embedded Tissues

**DOI:** 10.1371/journal.pone.0126762

**Published:** 2015-05-11

**Authors:** Felipe Vaca-Paniagua, Rosa María Alvarez-Gomez, Hector Aquiles Maldonado-Martínez, Carlos Pérez-Plasencia, Veronica Fragoso-Ontiveros, Federico Lasa-Gonsebatt, Luis Alonso Herrera, David Cantú, Enrique Bargallo-Rocha, Alejandro Mohar, Geoffroy Durand, Nathalie Forey, Catherine Voegele, Maxime Vallée, Florence Le Calvez-Kelm, James McKay, Maude Ardin, Stéphanie Villar, Jiri Zavadil, Magali Olivier

**Affiliations:** 1 Group of Molecular Mechanisms and Biomarkers, International Agency for Research on Cancer, Lyon, France; 2 Group of Genetic Cancer Susceptibility, International Agency for Research on Cancer, Lyon, France; 3 Subdirección de Investigación Básica, Instituto Nacional de Cancerología, México D.F., México; 4 Unidad de Biomedicina, FES-Iztacala, Universidad Nacional Autónoma de México (UNAM), México D.F., México; 5 Unidad de Genómica y Secuenciación Masiva (UGESEM), Instituto Nacional de Cancerología, México D.F., México; 6 Departamento de Patología Molecular, Instituto Nacional de Cancerología, México D.F., México; 7 Departamento de Epidemiología, Instituto Nacional de Cancerología, México D.F., México; 8 Unidad de Investigaciones Biomédicas en Cáncer, Instituto Nacional de Cancerología, Instituto de Investigaciones Biomédicas, Universidad Nacional Autónoma de México (UNAM), México D.F., México; 9 Departamento de Tumores Mamarios, Instituto Nacional de Cancerología, México D.F., México; Cleveland Clinic Lerner Research Institute, UNITED STATES

## Abstract

Triple negative breast cancer (TNBC), defined by the lack of expression of the estrogen receptor, progesterone receptor and human epidermal receptor 2, is an aggressive form of breast cancer that is more prevalent in certain populations, in particular in low- and middle-income regions. The detailed molecular features of TNBC in these regions remain unexplored as samples are mostly accessible as formalin-fixed paraffin embedded (FFPE) archived tissues, a challenging material for advanced genomic and transcriptomic studies. Using dedicated reagents and analysis pipelines, we performed whole exome sequencing and miRNA and mRNA profiling of 12 FFPE tumor tissues collected from pathological archives in Mexico. Sequencing analyses of the tumor tissues and their blood pairs identified *TP53* and *RB1* genes as the most frequently mutated genes, with a somatic mutation load of 1.7 mutations/exome Mb on average. Transcriptional analyses revealed an overexpression of growth-promoting signals (EGFR, PDGFR, VEGF, PIK3CA, FOXM1), a repression of cell cycle control pathways (TP53, RB1), a deregulation of DNA-repair pathways, and alterations in epigenetic modifiers through miRNA:mRNA network de-regulation. The molecular programs identified were typical of those described in basal-like tumors in other populations. This work demonstrates the feasibility of using archived clinical samples for advanced integrated genomics analyses. It thus opens up opportunities for investigating molecular features of tumors from regions where only FFPE tissues are available, allowing retrospective studies on the search for treatment strategies or on the exploration of the geographic diversity of breast cancer.

## Introduction

Triple negative breast cancer (TNBC), defined by the lack of expression of the estrogen receptor, progesterone receptor and human epidermal receptor 2 (HER2), is characterized by an aggressive clinical course, an earlier age of diagnosis, and lacks efficient treatment [1]. TNBC is a heterogeneous disease, both at the histopathological and molecular levels [2]. Histopathological subtypes range from ductal carcinoma, the most frequent phenotype, to rare phenotypes such as metaplastic, adenoid or medullary [3]. Transcriptomics analysis and microRNA (miRNA) profiling have identified up to four TNBC subtypes, the most frequent (80–90%) corresponding to the basal-like subtype first described in previous studies [2,4–6]. Whole genome sequencing efforts have shown that TNBC is defined by a predominance of *TP53* alterations that can be present in up to 80% of the cases, and by a large set of mutated genes occurring with minor mutation frequencies [7]. These studies have also revealed that TNBC can carry between just a handful to hundreds of somatic mutations [7, 8].

TNBC is associated with *BRCA1* germline mutations and has been reported to be more frequent in certain populations [9–11]. Indeed, studies conducted in the United States of America showed that women with TNBC are more likely to be of African and Hispanic descent and to live in socioeconomically deprived areas [12]. Studies in Mexican and African women showed prevalence from 25 to 55% [13,14] compared to 15% in Caucasian women [15]. In these populations, TNBC is also more prevalent in premenopausal compared to postmenopausal women [16]. A better understanding of the molecular heterogeneity and underlying biology of TNBC in these populations is thus essential to develop prevention and treatment strategies.

In countries where TNBCs are more prevalent, tumor samples are mainly accessible as formalin-fixed paraffin embedded (FFPE) archived tissues. The possibility to use these types of samples for comprehensive genomic analyses would allow a better characterization of the full spectrum of TNBC molecular features in these populations. Although recent developments have been made to adapt protocols and reagents to FFPE samples, performing advanced, multi-faceted molecular analyses such as genomics studies on this type of sample remains a technical challenge.

In this study, we assessed the feasibility of advanced molecular profiling of archived clinical samples of TNBC collected in Mexico. We performed transcriptomic (mRNA and miRNA) profiling and exome sequencing of 12 TNBC from Mexican women and showed that these integrated analyses can be achieved in archival FFPE samples. These molecular features were consistent with the basal-like subtype described in other populations.

## Materials and Methods

### Patients and samples

A retrospective series of 12 Mexican female patients diagnosed with primary TNBC (stages IIA-IIIB) at the National Cancer Institute of Mexico (INCAN) was selected based on the availability of tumor material in FFPE blocks (with tumor cellularity above 70%) and paired peripheral blood DNA. Patients were treatment naïve at the time samples were collected. They provided written informed consent for participation in the study. Mean age of patients was 48 years (range 30–64). Five patients were premenopausal and seven were postmenopausal. Disease stages were from IIA or IIIB. Average tumor size was 3.8 cm (range 1–15) with an average tumor cellularity of 76% (range 50–90) (S1 Table). Punch biopsies from the blocks were done to enrich for regions containing the highest proportion of tumor cells for DNA extraction. Samples of punched tumor tissue, peripheral blood DNA and tumor sections on slides were anonymized and shipped to the International Agency for Research on Cancer (IARC) for molecular analyses.

### Ethical approvals

This study was approved by the Ethics and Scientific committees of the National Cancer Institute of Mexico and by the IARC Ethics Committee.

### Nucleic acids extraction

Genomic DNA was extracted from FFPE tumor tissues using the QIAamp DNA FFPE Tissue Kit (Qiagen) following manufacturer’s instructions. Genomic DNA was isolated from peripheral blood with the Magna Pure System (Roche) following manufacturer instructions. The integrity of the material was verified by Bioanalyzer profiling (Agilent). Sample quantification was done with the Qubit dsDNA HS Assay Kit (Invitrogen). Total RNA (mRNA and miRNA) was extracted from FFPE tumor tissues with the RNeasy FFPE Kit (Qiagen) following manufacturer’s instructions.

### Whole gene expression profiling and differential gene expression analysis

Transcriptomic analysis of the 12 tumor samples was done with the FFPE-designed WG-DASL (Illumina) assay (assess 29,285 annotated transcripts) according to manufacturer’s instructions. Briefly, 200 ng of total RNA were reverse-transcribed into biotinylated cDNA, which was then primer-extended with the Assay Specific Oligos. The cDNA was then amplified with universal primers and hybridized to Illumina Human WG DASL HT Expression BeadChip arrays. The Illumina Genome Studio V2010.2 was used to obtain the signal values (AVG-Signal), with no normalization and no background subtraction. A technical duplicate was performed for all samples. The performance of hybridizations was evaluated by assessing the presence of outliers and the noise-to-signal ratios by calculating the ratio of centiles P95/P05 prior to normalisation for each sample. We defined outliers as samples with P95/P05 ratio <9.5. All samples were found to show a correct noise-to-signal ratio (P95/P05>9.6). The expression data have been deposited in NCBI's Gene Expression Omnibus [GEO:GSE62502] [17].

For differential gene expression analysis, two public datasets generated on the Illumina HumanHT-12 v3.0 beadChip, which contains 99.98% of the 29,285 probes of the Human WG DASL HT BeadChip were used as normal breast tissue controls: NCBI GEO GSE17072 and GSE32124 including five and 33 fresh frozen tissue samples, respectively. Identity and concordance of the array probes between these public datasets and the probes used in this study were verified by direct comparison using R. Genespring software GX 11.2 (Agilent, Santa Clara, USA) was used to perform quantile normalization on all datasets and differential expression analysis using the paired t-test method. Significance levels (p-values) were corrected using the Benjamini-Hochberg false discovery rate (FDR) method to correct for multiple hypothesis testing. Probes with a FDR-adjusted p-value of <0.01 and probes with a minimum of 2-fold and 1.5-fold change were considered significantly differentially expressed for the GSE32124 and GSE17072 datasets respectively. Genes for which probes showed contradictory direction of regulation were excluded from further analyses. The Database for Annotation, Visualization and Integrated Discovery (DAVID) v6.7 was used for classification of the differentially expressed genes according to biological and molecular processes. Gene Set Enrichment Analysis (GSEA) (Broad Institute) was used to define biologically relevant sets of genes based on experimentally validated gene sets deposited in the Molecular Signature Database (MSigDB). Only gene sets represented with a p-value of <0.01 were considered.

### miRNA profiling and differential expression analysis

Human TaqMan Low Density Arrays were used for profiling 754 mature miRNAs (TLDA A v2, TLDA B v3; Applied Biosystems) following manufacturer’s instructions. Briefly, 500 ng of total RNA (same aliquots as those used for whole gene expression) were reverse transcribed with the Megaplex RT Primers. cDNA was quantified using the 7900 HT Real-Time PCR system (Applied Biosystems). The signals obtained from the CT values were used for percentile shift normalization and correction using The Benjamini-Hochberg method in the Genespring software GX 11.2 (Agilent). For differential expression analysis, six normal fresh frozen breast tissues profiled with the same TLDAs (NCBI GEO accession number GSE35412) were used as control samples. MicroRNAs with a FDR- adjusted p-value of <0.01 and with a minimum of 2-fold change were considered as differentially expressed and were ranked by percentile values.

### PAM50 tumor subtype classification

From the 50 genes included in the PAM50 classifier, 49 were present on the DASL array. Expression values from these genes obtained in the microarray experiments were used to classify samples in the four subtypes defined with PAM50. Correlation of PAM50 centroids was computed on summarized values. Classification was considered as the centroid with the biggest correlation. Expression values of probes corresponding to the same gene were averaged and the correlation was computed on these values. P-value was calculated as the proportion of times that the maximum correlation to each centroid is greater than or equal to the maximum observed correlation in 100,000 resamplings of the unsummarized data.

### Integrated analysis of miRNA-mRNA relationships

The significantly modulated (up- or down-regulated) miRNAs and mRNA were used as input to the Ingenuity Pathways Analysis (IPA) suite (Ingenuity Systems, Redwood City, CA) for identifying potential miRNA-mRNA regulatory relationships. Only relationships defined as experimentally observed or high confidence predictions were considered. Predictions were based on data deposited in various databases (TargetScan, TarBase, miRecords, miRanda, DIANA-microT, miTarget, PicTar and PITA) taking into account stringent criteria such as strong evolutionary conservation, seed site number in the mRNA, site sequence context, thermodynamics parameters and pairing stability. From the list of interactions and networks found by the IPA suite (S10 Table), a reduced complexity network was built for each list (miRNAup/mRNAdn and miRNAdn/mRNAup), taking into account only interactions involving mRNA of genes involved in cancer (based on IPA disease annotations) and a miRNA connectivity score above 4. Networks were visualized using the Cytoscape 3.1.0 software. For the miRNAdn/mRNAup network, clusters were selected using the MCL algorithm of the ClusterMaker plugin using the default settings as reported elsewhere [18–20].

### Exome sequencing and somatic mutation analysis

Genomic DNAs from tumor tissue and from peripheral blood were used for library preparation with the 5500XL SOLiD Fragment Library Core Kit (Life Technologies) and exome capture with the TargetSeq Exome Kit (Life Technologies) with minor modifications. Single fragment sequencing was then performed with the SOLiD 5500XL platform (Life Technologies). Briefly 1.5–3 ug of DNA were sonicated with a Covaris S220 to 160 bp mean size (120–200 bp range) with the program: 10% duty cycle; intensity 5; 100 cycles per burst; 6 cycles of 60 seconds for blood DNA and 6 cycles of 100s for FFPE DNA; in frequency sweeping mode. Sheared fragments were end-repaired, size-selected (100–250 bp) using the Agencourt AMPure XP magnetic beads (Beckman Coulter), dA-tailed and ligated to P1-T and Barcode-T adaptors and amplified in 6–8 PCR cycles. Quality controls included quantitation of DNA after size-selection, ligation of adaptors and amplification of DNAs using the Qubit dsDNA HS Assay Kit and the Qubit 2.0 fluorometer, and DNA profiling of at the same steps using the High Sensitivity DNA Kit and the 2100 Bioanalyzer (Agilent). 62.5 ng of amplified material were pooled in batches of four and used for the exome capture with the TargetSeq Exome Enrichment kit which is designed to target 37.3 Mb of exomic sequence representing >98% VEGA, CCDS coding regions, and RefSeq exons. The pooled barcoded libraries were purified with Dynabeads M-270 Streptavidin (Invitrogen) and amplified by 6–8 PCR cycles and purified with Agencourt AMPure XP (Beckman Coulter). Emulsion PCRs were done with the SOLiD EZ Bead system (Lifetechnologies). Captured and emulsion amplified libraries were sequenced with the SOLiD 5500XL (1x75bp) using the Exact Call Chemistry (ECC) module to a calculated average coverage of 100X for tumors and 50X for blood DNA across targeted regions.

Exome data analyses (mapping and variant calling) were performed with LifeScope suite software (Lifetechnologies), using a combination of LifeScope targeted resequencing (TR) and low frequency variant detection (LFVD) workflows with the default parameters: (i) the color space reads generated by the SOLiD5500XL were mapped against the human genome reference version hg19 generating bam files which were then enriched for targets and good quality reads; (ii) single-nucleotide variants (SNVs) were called on enriched bam in both TR and LFVD workflows and small insertions and deletions (indels) were called on enriched bam in TR workflow. The LFVD aligned BAM files were submitted to the Sequence Read Archive [SRA: PRJNA261515]. The Variant Call Format (VCF) output files were annotated using the Annovar software including functional annotations as well as frequencies in known databases and in our custom SOLID catalogue of variants. Finally the VCF files were annotated using a custom “back-to-bam” script that added the number of reads found at each position with each allele and on each strand. Only variants present in both strands and in uniquely mapped reads were considered. Annotation of variants was done with ANNOVAR. Tumor-specific somatic mutations were obtained by subtracting tumor mutations found in the paired DNA blood sample. Somatic mutations were further filtered as follows. Exclusion criteria: mutations where the variant allele was found in less than two sequence read start sites; mutation in the context of an homopolymer tract of more than six repeated nt upstream or downstream; synonymous mutation; mutation with depth >800X (to eliminate false-positive variants over-represented by misalignment); allelic frequency >0.1% in either the 1000 Genomes project or the Whole Exome Sequencing Project (Washington University, which includes 6500 sequenced samples); allelic frequency >5% in the IARC generic catalogues of sequenced samples and a TCGA dataset of 650 samples (to eliminate systematic sequencing errors). Inclusion criteria: mutation found in an exonic or splice site; mutation not present in a duplicated genomic region; mutation predicted as deleterious by SIFT or PolyPhen-2.

To further select potentially pathogenic alterations, mutations were annotated as driver events if present in genes (1) defined as tumor suppressors or oncogenes in GSEA (Broad Institute) database, (2) considered as drivers by Vogelstein et al [21], (3) identified as significantly mutated genes in the five studies of breast cancer available in the TCGA database. Collectively, these databases contain more than 6,000 sequenced tumors. All filtered variants were verified by manual inspection of the BAM files using the Integrated Genome Viewer (Broad Institute). Recurrent somatic mutations were confirmed experimentally by sequencing both the tumor and blood DNA samples with the Ion Torrent PGM platform using targeted amplicon sequencing at 500X minimum coverage, following manufacturer’s instructions.

## Results

### Deregulated transcriptional programs

**Fig 1:**
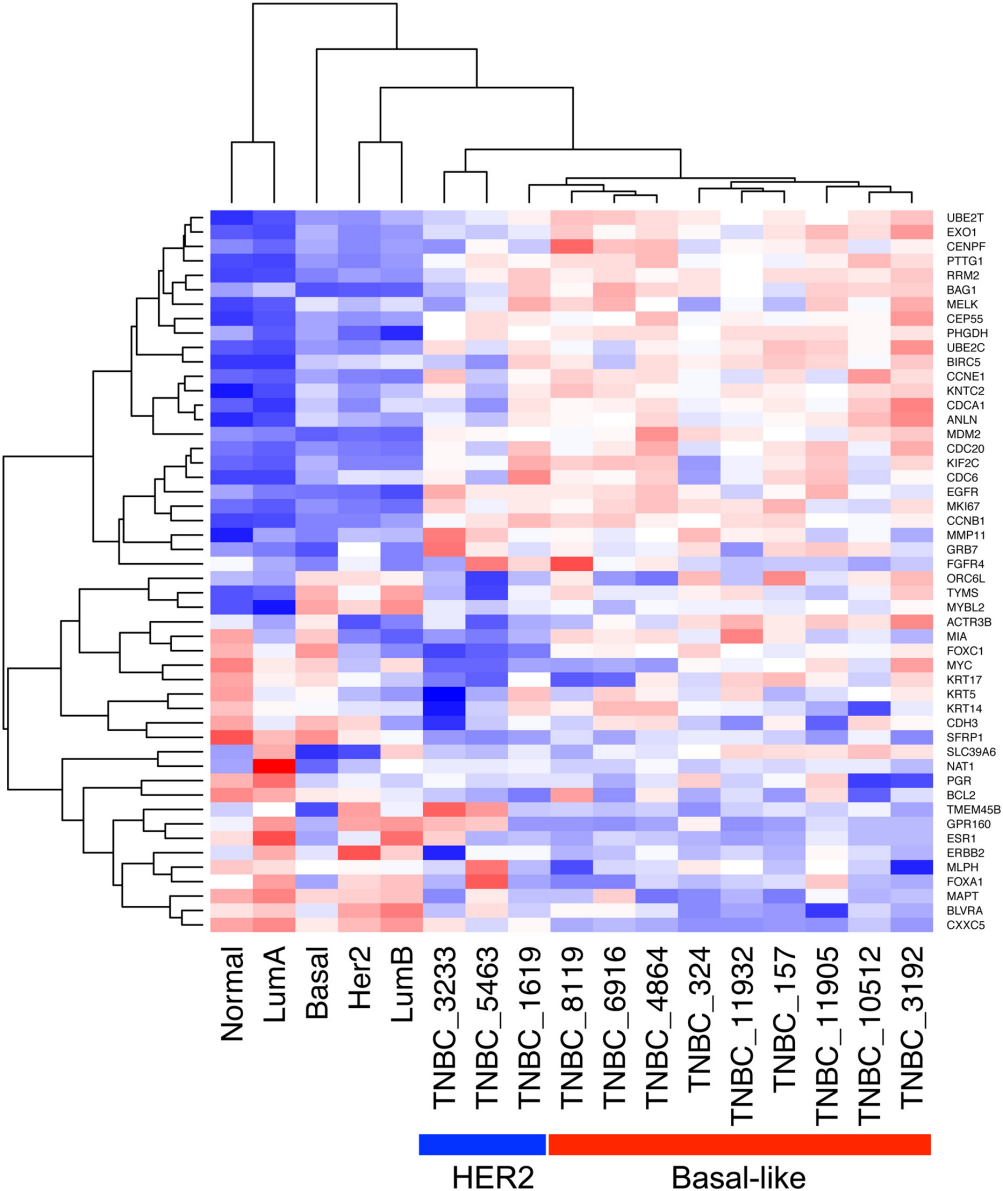
PAM50 classification of TNBC samples. The hierarchically-clustered normalized expression values of the PAM50 classifier genes is shown for the 12 triple negative breast cancers (TNBCs) analyzed and the five centroids. The samples were classified according to their correlation with the PAM50 centroids. Red and blue boxes represent overexpressed and down-regulated genes, respectively.

**Fig 2:**
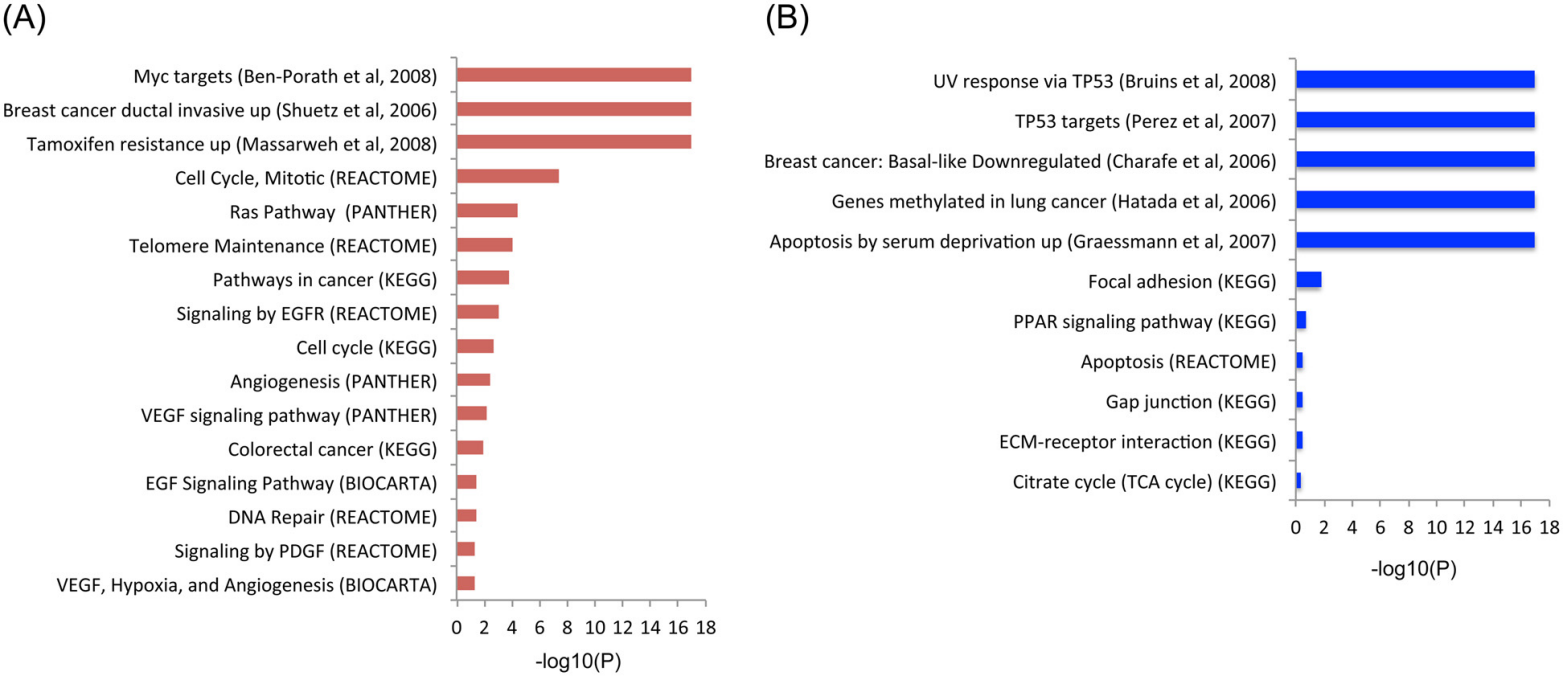
Enriched pathways from the differential gene expression analysis. (A) Up-regulated pathways from KEGG, PANTHER, REACTOME and Chemical and Genetic perturbations (GSEA). (B) Down-regulated pathways from the same sources. Enrichment is shown as-log10(P) values.

To characterize the transcriptional programs expressed in TNBC tumors from Mexican women, archived tumor samples were analyzed by whole gene expression profiling using FFPE specific reagents for RNA extraction and analysis (see Methods). The FFPE-designed WG DASL HT (Illumina) expression assay covers 20,727 unique genes. We first classified samples using the PAM50 classifier and found that 75% (9/12) of the tumors belonged to basal-like molecular subtype while 25% corresponded to the HER2 subtype (Fig 1, S2 Table). Differential gene expression analysis (DGEA) was then performed using two independent public control datasets (obtained on the same type of analysis platform) of normal breast tissues as paired normal tissues were not available from the tissue archive (see Materials and Methods). Using the first control set (GSE32124, 33 samples of normal tissues), 7,459 of 20,727 (36.0%) genes were differentially expressed in the tumor tissues, with 18.0% and 17.9% of genes up- and down-regulated respectively (S3 and S4 Tables). Using the second control set (GSE17072, five samples of normal tissues), 1,590 of 20,727 (7.7%) genes were found differentially expressed with 4.4% and 3.3% of genes up- and down-regulated respectively (S5 Table). There was a near 90% overlap between these two independent analyses, with only one gene related to cancer that had contradictory direction of regulation (S1 Fig). Therefore, considering the stringency of the statistics used (T-test p = 0.01, FDR = 0.01), the substantial overlap between both analyses, and the fact that the larger number of controls may resemble more accurately the transcriptional nature of the normal breast tissue, we used results obtained with the GSE32124 dataset (33 controls). This DGEA analysis showed that, among genes expected to be overexpressed in the TNBC phenotype, *MKI67*, *TOP2A*, *CCNE1*, *CCNE2*, *EGFR*, *FGFR1*, *FGFR2*, *VEGFA*, *HIF1A*, *ARNT*, *FOXM1* and the BRCA1-repressor *ID4* were found up-regulated (S3 Table). Interestingly, *MYC* mRNA was not up-regulated, but there was a significant enrichment of 37 overexpressed genes in the gene set corresponding to cytogenetic band 8q24 that includes *MYC* gene (S6 Table). Significant enrichment was also found for other cytogenetic bands associated with breast cancer, including 1q21, 1q22, 1q32, 3q23 and 3q28 (S6 Table). Among other up-regulated genes, those that contain transcription-binding sites for *MYC*, *MAX*, *MYB*, *ELK1*, *ETS1*, *ETS2*, *ETV7*, *MAF* and *E2F* were significantly enriched (S7 Table). Significant enrichment was also found for growth promoting and tumor progression pathways such as *EGFR*, *MYC*, *VEGFR* and *E2F*; for biological processes linked to proliferation such as telomere maintenance, cell cycle, DNA repair, DNA replication chromosome organization; and for gene sets associated with ductal invasive breast cancer and exposure to *EGF* or *MYC* activity (Fig 2A). Interestingly, the isoforms of the apolipoprotein B mRNA-editing enzyme catalytic polypeptide-like (*APOBEC3A*, *APOBEC3B* and *APOBEC3H*), implicated in breast cancer carcinogenesis, were also up-regulated (S3 Table). In addition, 10 of the 14 genes associated with the Basal-like Immune Activated (BLIA) subtype of TNBC recently reported by Burstein et al., where up-regulated, including *CXCL11*, *RARRES1*, *GBP5*, *CXCL10*, *CXCL13*, *LAMP3*, *STAT1*, *CTLA4*, *TOP2A*, *LCK* [22].

Consistent with the triple negative phenotype, *ESR1* and *ERBB2* were down-regulated (although *PGR* levels did not show a statistically significant down-regulation) (S4 Table). Within down-regulated genes, there was significant enrichment for *TP53* targets, genes down-regulated in basal-like breast cancer, respiratory electron transport and cell adhesion pathways, *MYC* repressors, *BRCA*, *CHEK2* and *ATM* networks (Fig 2B and S4 Table).

### miRNA expression analysis and networks of miRNA:mRNA connectivity

The expression profile of 754 miRNAs was determined in the 12 tumor samples using TaqMan arrays. To identify deregulated miRNAs, a control dataset of six normal tissues (deposited in GSE35412) obtained with the same analysis platform was used for differential expression analysis (see Materials and Methods). We found a pattern characterized by high expression of oncogenic miRNAs and repression of tumor suppressor miRNAs (S8 and S9 Tables). The clusters of highest enrichment in the up-regulated miRNAs were the miR-1283 (7 miRNAs), miR-1185 (6 miRNAs) and miR-17 cluster (5 miRNAs). Consistent with its polycistronic nature, all members of the miR-17-92 oncogenic cluster showed co-regulation, with the exception of miR-19a. Furthermore, the miR-17 and miR-548 families were the most enriched ones with five and seven members involved, respectively. Overall, the top five up-regulated miRNAs were miR-624, miR-339-5p, miR-191 and miR-651. The top five up-regulated oncogenic miRNAs were miR-93, miR-20a, miR-214, miR-146b and miR-92a (percentile rank of overexpressed miRNAs 92.9, 91.4, 84.3, 75.8 and 68, respectively). Strongly down-regulated tumor suppressing miRNAs included miR-1, miR-34c, let-7a, let-7b, miR-127, with a percentile rank of inhibition of 96.2, 84.2, 69.1, 61.6, 59.3, respectively. Other down-regulated tumor suppressors were let-7c, miR-101, let-7e, miR-125b, miR-141, miR-126, miR-34a, miR-34c and miR-200a.

**Fig 3:**
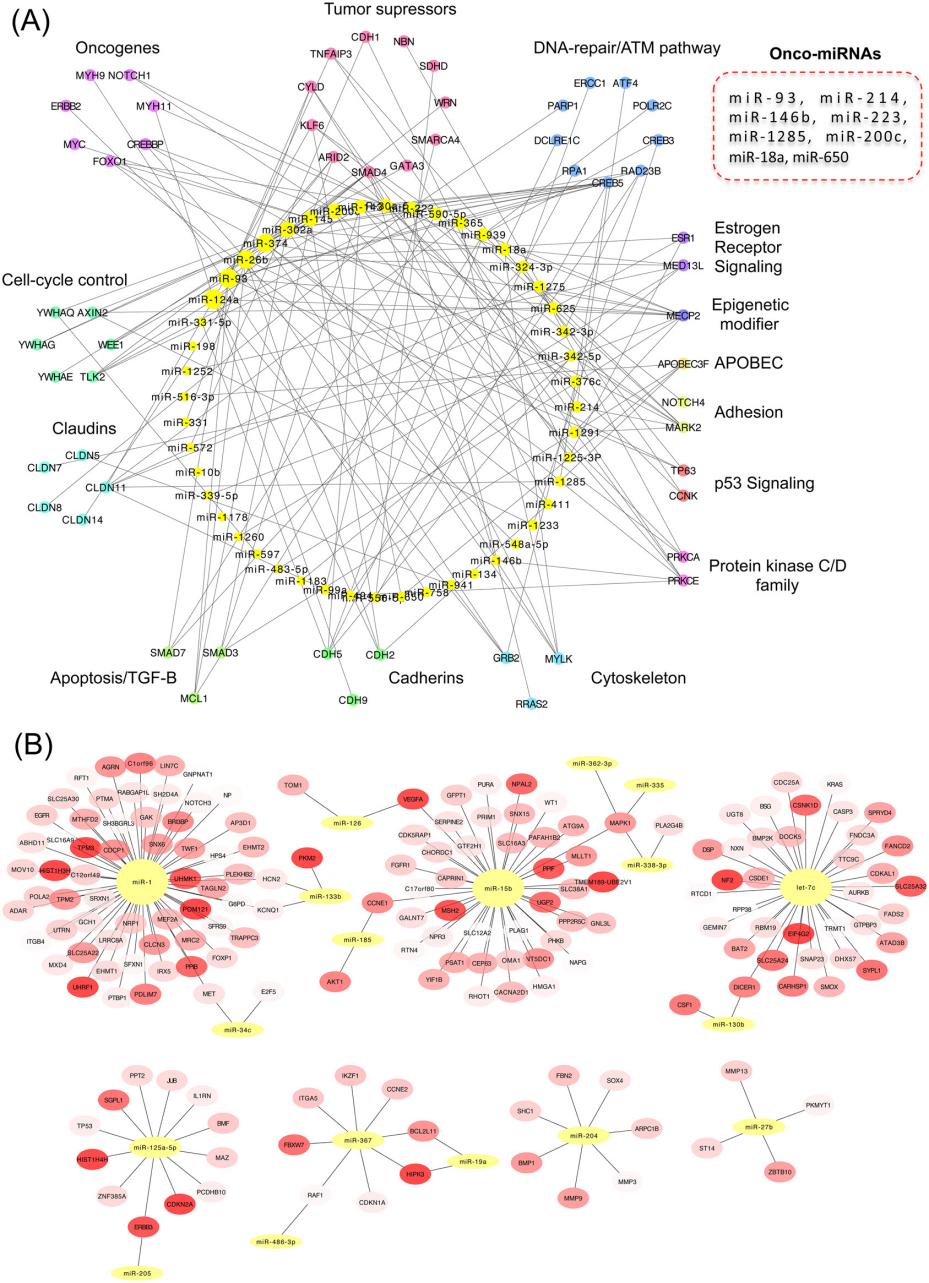
Networks of connectivity between miRNA and mRNA. (A) Networks of (A) up-regulated miRNAs and down-regulated mRNAs, (B) down-regulated miRNAs and up-regulated mRNAs, identified in the TNBC samples (only high confident interactions and genes involved in cancer were selected).

In order to identify potential networks of post-transcriptional regulation of gene expression, we analyzed inverse relationships of miRNAs and their target mRNAs in the differentially expressed paired profiles focusing on genes involved in cancer (see Materials and Methods). We found a repressive network composed of 53 overexpressed miRNAs and 68 down-regulated genes (Fig 3A, S10 Table). The repressed genes were significantly enriched for the ontology terms “regulation of apoptosis” and “tight junction”. Tumor associated miRNAs present in this network included miR-18a, miR-93, miR-146b, miR-200c, miR-214, miR-223, miR-650 and miR-1285, collectively repressing 9 tumor suppressors genes (including *ARID2*, *ATM*, *CYLD*, *KLF6*, *NBN*, *SDHD*, *SMAD4*, *SMARCA4*, *TNFAIP3*), nine oncogenes (including *BCL2*, *CREB1*, *CREBBP*, *FOXO1*, *MYC*, *MYH11*, *MYH9*, *NOTCH1*), growth inhibitors (*BMPR2*, *BTG2*, *BTG3*, *LITAF* and *PA2G4*), chromatin remodelers, six claudin genes (*CLDN11*, *CLDN14*, *CLDN5*, *CLDN7*,*CLDN8*, *CLDND1*) 11 DNA-repair/ATM pathway genes (*ATF4*, *CREB3*, *CREB5*, *DCLRE1C*, *ERCC1*, *PARP1*, *POLR2C*, *RAD23B*, *RAD51*, *RPA1*, *XRCC5*) and six proapoptotic genes (*BAK1*, *BCL2L1*, *MCL1*, *SMAD3*, *SMAD5*, *SMAD7*). Interestingly, the transcripts of the estrogen receptor (*ESR1*), *HER2* (*ERBB2*) and E-cadherin (*CDH1*) were part of this suppressive network (Fig 3A). In the reverse association, there were 32 connected clusters of down-regulated miRNAs and up-regulated target mRNAs. Seven of these clusters harbored four or more genes (Fig 3B) and showed a significant functional enrichment for kinase and cell cycle activity, and for focal adhesion pathways. The clusters with the highest number of mRNAs included tumor suppressing miRNAs such as miR-1 (connected to 63 mRNAs), miR-15b (linked to 43 mRNAs), let-7b (connected to 37 mRNAs), miR-125a (connected to 13 mRNAs) and miR-204 (linked to 7 mRNAs) (Fig 3B). Oncogenes present in these clusters included *EGFR*, *FGFR1*, *HRAS*, *KRAS*, *FOXP1*, *HMGA1*, *MET*, *MLLT1*, *NOTCH2*, *PAFAH1B2*, *PICALM*, *PLAG1*, *RUNX1*, *TPM3*, and five growth factors (*BMP1*, *CSF1*, *IL1RN*, *TGFB3* and *VEGFA*), some of which are typically overexpressed in TNBC (Fig 3B).

### Landscape of somatic mutations

**Fig 4:**
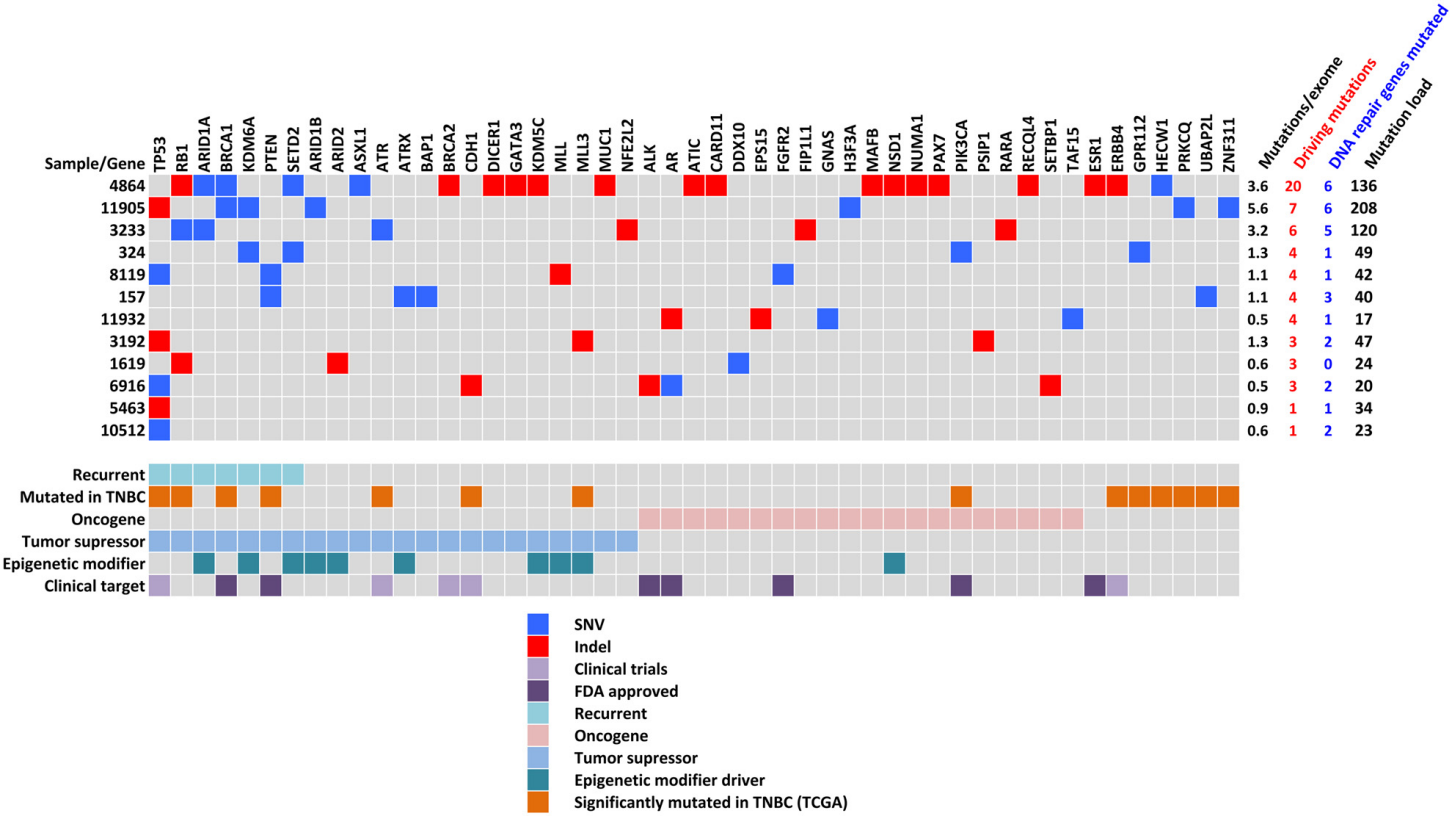
Summary results of whole exome sequencing for all matched samples (n = 12). Top panel, details of mutations found in tumors in each specific samples (blue squares are single-nucleotide variants (SNVs) and red squares are insertions and deletions (Indels). Right panel shows the mutations per exome, the amount of mutations in driving and DNA repair genes and total number of mutations per tumor. Lower panel shows gene annotations derived from present results and other studies: Recurrent gene mutated in more than one sample in present series; Mutated in triple negative breast cancer (TNBC) [7], significantly mutated in TNBC from TCGA [49,50]; Oncogenes and tumor suppressors class derived from references [21,51]; Epigenetic modifiers class derived from references [52,53]; Clinical target, genes that are targets of drugs in clinical trials or approved by FDA. (http://www.fda.gov/Drugs/InformationOnDrugs/ApprovedDrugs/default.htm).

The 12 tumors and their paired blood DNA were sequenced by whole exome sequencing at a mean coverage of 123x (range, 54–269) and 65x (range, 56–82) respectively (Table 1). Using a dedicated bioinformatics pipeline combining high mapping and calling stringency, and exclusion of platform-specific sequencing errors (see Materials and Methods), we found a mean frequency of 1.7 mutations/exome Mb (range, 0.46–5.56), which is concordant with previous studies in TNBC [7,23]. To define potential driving mutations, we applied a filtering approach based on mutation position (splicing sites, exonic), nature of the mutation (non-synonymous with predicted deleterious impact on protein), its genomic context, absence in human populations without cancer, and the biological relevance of the affected gene (see Materials and Methods). With this strategy, we found samples with 1 to 20 driving mutations with a mean of 5.25 driving mutations per sample (Fig 4; S11 Table). Two tumors with only one driving mutation had *TP53* affected. Recurrent driving genes with pathogenic mutations were *TP53* (54%), *RB1* (27%), *ARID1A* (18%), *BRCA1* (18%), *KDM6A* (18%), *PTEN* (18%) and *SETD2* (18%). Other relevant cancer genes that were mutated in only one sample included *ATR*, *ATX*, *BRCA2*, *CDH1*, *GATA3*, *PAX7*, *PIK3CA* and *ESR1*. Thirty percent of the mutated driving genes were druggable with 91 anti-neo-neoplastic agents, of which 18 are drugs approved by the Food and Drug Administration (FDA) (S12 Table). Interestingly, pathogenic mutations were found in 19 DNA repair genes and tumors with higher numbers of mutations in DNA repair genes showed higher mutation load (Fig 4; S2 Fig). Given that we found three isoforms of the *APOBEC3* cytidine deaminase overexpressed in these tumors, we looked for the expected signature of the APOBEC3 family of enzymes. The APOBEC signature is characterized by C to T and C to G mutations in a T**C**W sequence context [24–26]. In our set of tumors the main mutation type was C to T with a majority occurring in the context of 5’-T**C**T-3’, resembling the APOBEC signature. Mutations with this signature were found in cancer driving genes such as *ATRX*, *FGFR2*, *SETBP1* and *TP53*. However, there was no correlation between APOBEC mRNA expression and the total number of C to T transitions in the context of T**C**W at the sample level.

**Table 1:** Whole exome sequencing summary statistics.

Metric	Value (range)
Tumors-normal pairs sequenced	12
Total sequenced (GB)	198.43
Mean fold tumor target coverage (range)	123x (54–269)
Mean fold normal target coverage (range)	65x (56–82)
Mean somatic mutation rate per megabase (range)	1.7 (0.46–5.56)

### Summary integrated analysis

#### TP53 pathway

**Fig 5:**
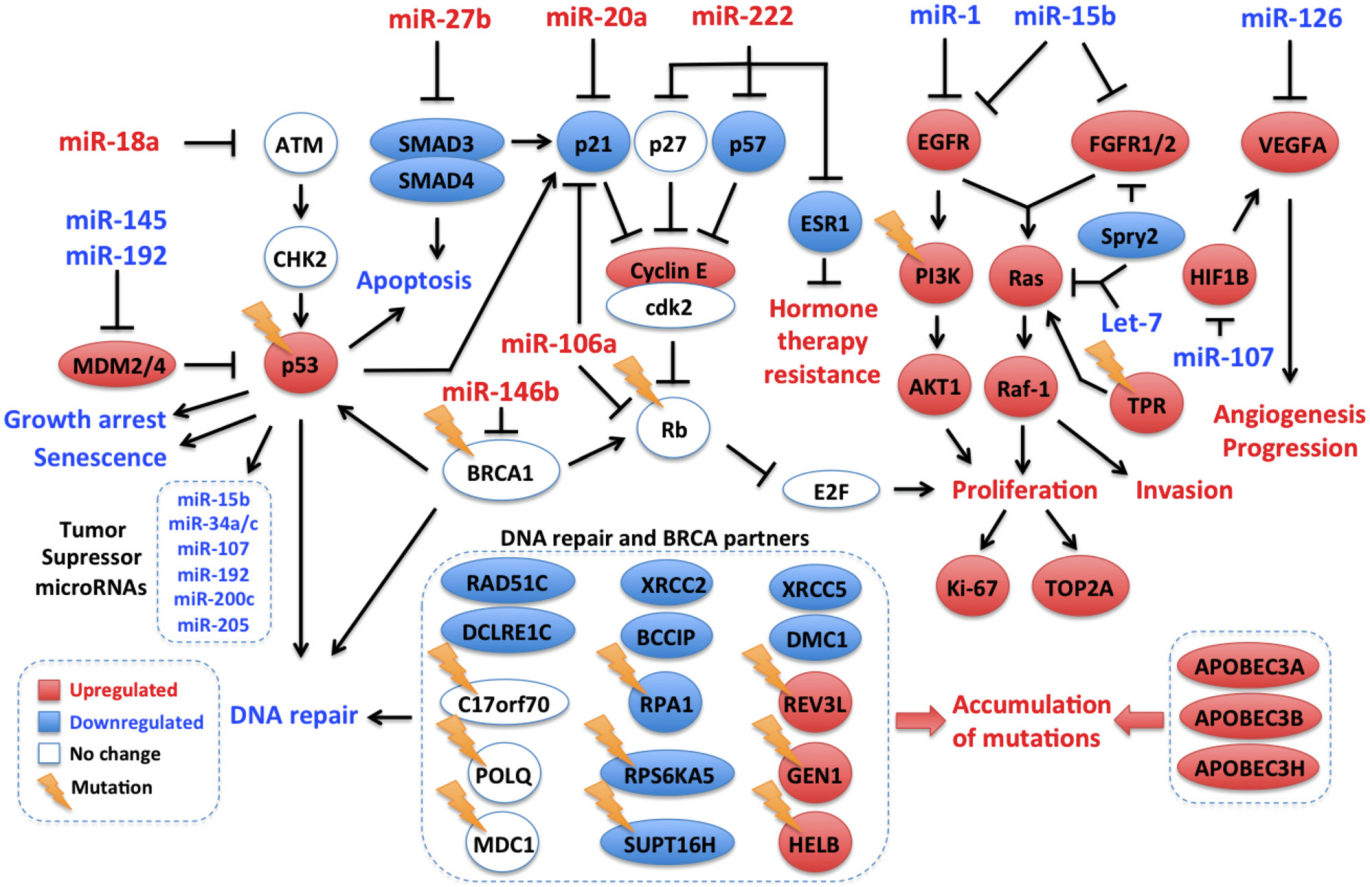
Integrated molecular portrait of triple negative breast tumors from Mexican women using archived clinical samples. Data from whole exome sequencing and miRNA and mRNA expression experiments were integrated. Tumor suppressor pathways and DNA repair genes are repressed through different mechanisms, while tumor growth and progression pathways and genes related to endogenous mutation promotion exhibit up-regulation. Gene, miRNA and Pathway expression levels are depicted in red for up-regulation and blue for down-regulation; genes without differential expression are marked in white. Mutations are shown as lightning icons.

*TP53* was found mutated in 50% of the samples with a high proportion of truncating mutations (50%; Fig 4). Evidence for *TP53* inactivation by other mechanisms was also found. Indeed, the p53 inhibitors *MDM2* and *MDM4* were up-regulated at the mRNA level, and several miRNA acting downstream of p53 or impacting p53 were deregulated. A set of onco-suppressive microRNAs with p53 binding sites in their promoters were down-regulated. These microRNAs, that included miR-15b, miR-205, miR-34a, miR-34c, miR-192, miR-200a and miR-107 target p53 inhibitors (such as *MDM2*), cell cycle genes (*CDK4/6*, *E2F*, *CCNE1/2*, *CDC7*), oncogenes (*MET*), growth factors (*IGF1*), antiapoptotic genes (*BCL2*), metabolic genes (*LDHA*), stemness genes (*NANOG*, *SOX2*, *KLF4*, *OCT4*, *CD44*), proangiogenic genes (*ANRT*) [27–29] or genes involved in proliferation and metastasis (*LAMC1*). miR-449b and miR34 which block the p53 repressors *SIRT1* and *HDAC1* were down-regulated. miR-1285, which is a direct repressor of TP53 was overexpressed [30]. Finally, The MDM-blocking miRNA miR-192 was down-regulated (Fig 5).

#### PIK3CA/PTEN pathway

Only three samples showed direct activation of the *PIK3CA* pathway, with one sample (8%) carrying an activating mutation in *PIK3CA* (p.H1047L) and two samples carrying *PTEN* inactivating mutations. However, there was evidence for global activation of this pathway in the miRNA/mRNA expression analyses (Fig 5). Indeed, key *PIK3CA* effectors were overexpressed (*AKT1*, *PIK3CB*, *PIK3CD*, *PTK2*, *KRAS*, *CD19*, *HSP90AA1* and the integrins *ITGA1*, *ITGA5*, *ITGA8*, *ITGB4*, *ITGB6* and *ITGB8*), and the PTEN-targeting miRNAs miR-21, miR-222 and the AKT downstream targets *MDM2*, *NOS3*, *YWHAZ* were up-regulated. In addition the *FOXO1*, *BAD*, *CDKN1A*, *CDKN1B* genes, which encode proteins repressed by *AKT* were found down-regulated, as well as the *AKT* inhibitors miR-125b, miR-149, miR-184, miR-708, *PPP2CA*, *PPP2R1A*, *PPP2R1B* and *PPP2R5E* (S3 Fig).

#### RB1 pathway

An overall down-regulation of the Rb pathway was apparent, with mutations in *RB1* in three samples and global up-regulation of *MDM2* (RB1 repressor) and of RB1-targeting miRNAs (miR-215, miR-106a, miR-17, miR-20a, miR-93, miR-215, miR-21).

#### DNA repair pathway

Mutations in DNA repair genes were found in all samples but one, including four deleterious somatic mutations in *BRCA1*/*2* genes. Although no change was observed at mRNA level in BRCA genes, some of their important molecular partners were down-regulated including *BCCIP*, *RAD51C*, *DMC1*, *DCLRE1C* (Artemis), *XRCC2*, *XRCC5* and *RPA1* (Fig 5). Furthermore, the BRCA1-targeting miR-146b was among the top up-regulated miRNAs, which may result in BRCA1 repression through translation inhibition [31]. These results suggest a low capacity for genome maintenance and may thus reflect a genomic instability state in these tumors [32–34].

#### FOXM1 pathway

Consistent with other TNBC studies, *FOXM1* mRNA was overexpressed in these tumors and several *FOXM1* transcriptional targets were found up-regulated (*AURKB*, *BIRC5*, *CCNB1*, *CCNB2*, *CENPA*, *CENPF*, *NEK2*, *SKP2*, *XRCC1* and *MMP9)*. Furthermore, *FOXM1*, the inducers *RAF1*, *HIF1A*, *CCNA*, *CCNB* and *CCNE* were overexpressed, while the repressors miR-149 and miR-186 were down-regulated (S4 Fig). There was thus evidence for *FOXM1* pathway activation in these tumors.

#### Epigenetic modifiers

Interestingly, we found a total of 14 mutations in chromatin remodeling genes, including recurrent mutations in *ARID1A*, *KDM6A* and *SETD2* (Fig 4). Additionally, the expression levels of several genes with epigenome regulatory activity were altered, including 51 up-regulated and 23 down-regulated (S13 Table).

#### Invasiveness

Invasive/EMT-like phenotype has been reported in TNBC and could reflect the metastatic nature of these tumors. Although regulators of invasiveness such as Snail, Slug and Twist were not found deregulated, molecular markers like *MMP9* and Fibronectin and miRNA related to invasion (miR-222) were up-regulated, while *CDH1* was repressed. These results may reflect an invasive-like signature.

## Discussion

This study represents the most comprehensive genomic analysis performed on clinical FFPE samples of TNBC from an ethnically homogenous cohort of Latin American women. Combining whole exome sequencing, miRNA and mRNA transcriptomic profiling, we found a gene expression program and a profile of somatic mutations consistent with findings reported in recent integrated genomic analyses of large series of high quality breast cancer samples, including two studies focused on TNBC [7,35]. Although we analyzed a small set of samples (12 tumors), these samples have been carefully selected as molecularly homogeneous TNBC based on IHC analyses with more than 70% tumor cellularity. Our transcriptional analysis found that 9 out of 12 samples (75%) had an expression pattern of basal-like breast cancer (based on the PAM50 gene signature). These findings are thus consistent with the fact that basal-like subtype of breast cancer is found in 70–80% of TNBC [8]. Moreover, our samples may be more similar to the immune-activated basal-like subtype of TNBC that has recently identified in several sample sets [22].

The tumors analyzed harbored one to 20 driving mutations per tumor. *TP53* was the most frequently mutated gene, with a high proportion of truncating mutations, a feature previously described in basal-like breast cancer [36]. *RB1* was the second most frequent hit as reported before [7,35]. *PIK3CA* gene, which is frequently mutated in breast cancer, except in the TNBC/basal-like subtype, was also found rarely mutated (in 1/12, 8.3% of samples). Overall, half of the samples (6/12) carried genetic alterations potentially actionable by currently available drugs approved by the FDA. Deregulated transcriptional programs were characterized by growth and tumor promoting signals, with in particular the overexpression of EGFR, PDGFR and VEGF pathways, recognized as some of the main pathways driving TNBC [7,37]. The combined analysis of mutations and transcriptional programs showed that well-recognized pathways deregulated in basal-like tumors were found deregulated in these set of TNBC tumors (see Summary integrated analysis section and Fig 5). Thus, no specific molecular characteristics were found in these series of tumors, suggesting that TNBC in Mexican women develops through similar mechanisms to TNBC in other populations. It is of note that our analysis was focused on genes involved in cancer pathways and on well-defined molecular interactions, as our primary aim was to assess whether the major molecular alterations detected in this series of tumors matched the one described in other TNBC cases. However, this study shows that TNBC oncogenic molecular programs converge in key deregulated pathways that may be targeted, not only by chemotherapeutic agents and protein-targeting drugs, but also oncogenic miRNA blockers.

One of the challenging limitations of our study was the lack of normal tissue for the analysis of mRNA and miRNA differential expression. We circumvented this issue by employing public expression data of normal breast tissue obtained on the same or similar arrays as the one used here and by applying high stringency statistics, an approach that has been successfully applied in other breast cancer studies [38,39]. For the DGEA with mRNA we compared the performance of two datasets and found a high degree of concordance and no notable difference in the sense of regulation of the genes. For miRNA analysis, we could identify only one control set. Despite this limitation, the overall pathway and miRNA analyses confirmed expected molecular alterations found in other breast cancer and TNBC genomic studies, and corroborated the histological classification, which collectively reinforced the validity of our analysis.

While other studies showed the possibility of using FFPE samples for Next-Generation Sequencing (NGS) analysis [40,41], this remains challenging. For mRNA analysis we used reagents specially dedicated to FFPE samples that allow the detection of molecules in partially degraded samples [42,43]. In contrast, the stability of miRNA in FFPE samples has been described to be high in different studies [44,45] and we used a classical TaqMan platform that has been validated for FFPE samples [46,47]. Tissue fixation with formalin is known to increase C to T substitution artifacts during the PCR steps. In breast cancer, the up-regulation of the APOBEC3 enzymes has been identified as a major source of C to T mutations within the TCW sequence context [48]. In our analysis, although we found an overall up-regulation of *APOBEC3B*, *APOBEC3A* and *APOBEC3H* mRNA and a mutation signature enriched for C to T transitions, the level of mRNA expression of APOBEC members at the sample level did not correlate with the overall mutation load or the abundance of C to T mutations. Whether potential C>T artifacts due to the FFPE origin of the samples may have masked APOBEC mutation signature thus remains unclear. It is of note that, although we could not test all mutations in validation experiments due to lack of sufficient material, we could validate 20/22 (91%) mutations in recurrently mutated genes using targeted resequencing with the Ion Torrent (data not shown). Thus, although mutation confirmation is important for interpreting sequencing data from FFPE material, our work confirms the feasibility of using these biospecimens for NGS analysis. It is expected that technology developments will soon improve the quality of sequencing obtained with these samples.

## Conclusions

Our results show that an integrated molecular analysis including exome and miRNA:mRNA transcriptome is feasible on archival FFPE samples. The possibility to use FFPE material, which represents the most frequent sample type in biorepositories, is of broad interest since it opens the opportunity to perform large retrospective studies on treatment outcomes, to investigate rare tumor types and to explore the geographic diversity of breast cancer.

## Supporting Information

S1 Fig Differentially expressed genes against different control datasets. Differential expression analysis was done using the GSE32124 and GSE17072 GEO public datasets. The statistical conditions used, the number of differentially expressed genes and genes with contradictory direction of regulation are shown.
(PDF)

S2 Fig DNA repair genes mutated and sample mutation load. For each sample the burden of somatic mutations was plotted against the total number of DNA repair genes with pathogenic mutations. Pearson correlation is shown (n = 12).
(PDF)

S3 Fig Molecular alterations in PI3K pathway. Genes with pathogenic somatic mutations and differentially expressed genes and miRNAs that regulate PI3K pathway are shown. Gene, miRNA and Pathway expression levels are depicted in red for up-regulation and blue for down-regulation; genes without differential expression are marked in white. Mutations are shown as lightning icons.
(PDF)

S4 Fig Molecular alterations in FOXM1 pathway. Differentially expressed genes and miRNAs that regulate FOXM1 pathway are shown. Gene, miRNA and Pathway expression levels are depicted in red for up-regulation and blue for down-regulation; genes without differential expression are marked in white.
(PDF)

S1 Table Patients and sample characteristics. (XLSX)

S2 Table PAM50 tumor subtype classification. (XLSX)

S3 Table Probes of genes up-regulated in DGEA with GSE32124 (33 controls). (XLSX)

S4 Table Probes of genes down-regulated in DGEA with GSE32124 (33 controls). (XLSX)

S5 Table Genes up- and down-regulated in DGEA with GSE17072 (5 controls). (XLSX)

S6 Table Up-regulated genes enriched in cytogenetic bands. (XLSX)

S7 Table Transcription factor binding sites enriched in up-regulated genes. (XLSX)

S8 Table MicroRNAs up-regulated. (XLSX)

S9 Table MicroRNAs down-regulated. (XLSX)

S10 Table Integrated analysis of miRNA-mRNA relationships. (XLSX)

S11 Table Somatic mutations identified by whole exome sequencing. (XLSX)

S12 Table Genes with somatic mutations targeted with FDA-approved drugs (data base in: http://www.fda.gov). (XLSX)

S13 Table Molecular alterations in epigenetic modifier genes. (XLSX)
