# Supplementary material for: Revealing the Molecular Portrait of Triple Negative Breast Tumors in an Understudied Population through Omics Analysis of Formalin-Fixed and Paraffin-Embedded Tissues
Source: PLoS One. 2015 May 11;10(5):e0126762. doi: 10.1371/journal.pone.0126762 (PMC4427337; doi:10.1371/journal.pone.0126762)
Supplement: S1 Fig — Differential expression analysis was done using the GSE32124 and GSE17072 GEO public datasets. The statistical conditions used, the number of differentially expressed genes and genes with contradictory direction of regulation are shown. (PDF) [file pone.0126762.s001.pdf]

S1 Figure. Differentially expressed genes against different control datasets. Differential expression analysis was done using the GSE32124 and GSE17072 GEO public datasets. The statistical conditions used, the number of differentially expressed genes and genes with contradictory direction of regulation are shown.

Reference	Controls	Diff expressed (%)	Upreg(%)	Downreg (%)	fold-change	ttest p	FDR
GSE32124	33	7459 (36.0)	3741 (18.0)	3718 (17.9)	2	0.01	0.01
GSE17072	5	1590 (7.6)	909 (3.9)	681 (3.3)	1.5	0.01	0.01

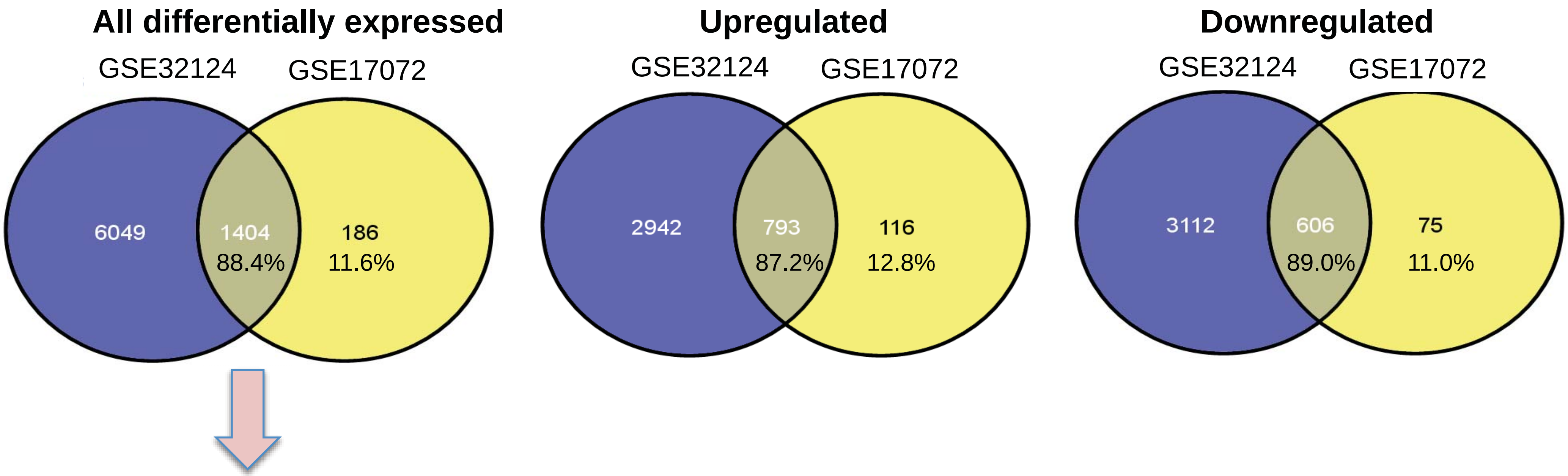

Contradictory regulation

- ACAD8
- GTF2A1
- PPP1CB
- RARA
- VAPA
