# Supplementary material for: Revealing the Molecular Portrait of Triple Negative Breast Tumors in an Understudied Population through Omics Analysis of Formalin-Fixed and Paraffin-Embedded Tissues
Source: PLoS One. 2015 May 11;10(5):e0126762. doi: 10.1371/journal.pone.0126762 (PMC4427337; doi:10.1371/journal.pone.0126762)

S2 Figure. DNA repair genes mutated and sample mutation load. For each sample the burden of somatic mutations was plotted against the total number of DNA repair genes with pathogenic mutations. Pearson correlation is shown (n = 12).

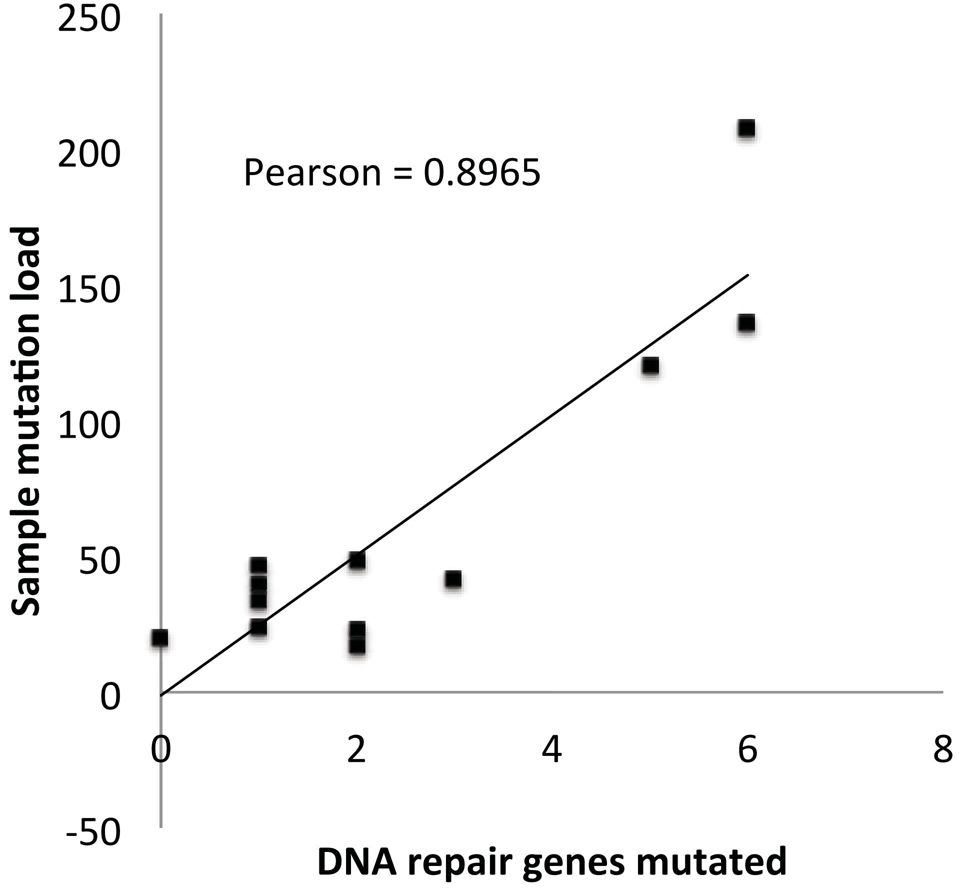

Supplement: S2 Fig — For each sample the burden of somatic mutations was plotted against the total number of DNA repair genes with pathogenic mutations. Pearson correlation is shown (n = 12). (PDF) [file pone.0126762.s002.pdf]
