# Supplementary material for: Revealing the Molecular Portrait of Triple Negative Breast Tumors in an Understudied Population through Omics Analysis of Formalin-Fixed and Paraffin-Embedded Tissues
Source: PLoS One. 2015 May 11;10(5):e0126762. doi: 10.1371/journal.pone.0126762 (PMC4427337; doi:10.1371/journal.pone.0126762)

**S3 Figure. Molecular alterations in PI3K pathway.** Genes with pathogenic somatic mutations and differentially expressed genes and miRNAs that regulate PI3K pathway are shown. Gene, miRNA and Pathway expression levels are depicted in red for up-regulation and blue for down-regulation; genes without differential expression are marked in white. Mutations are shown as lightning icons.

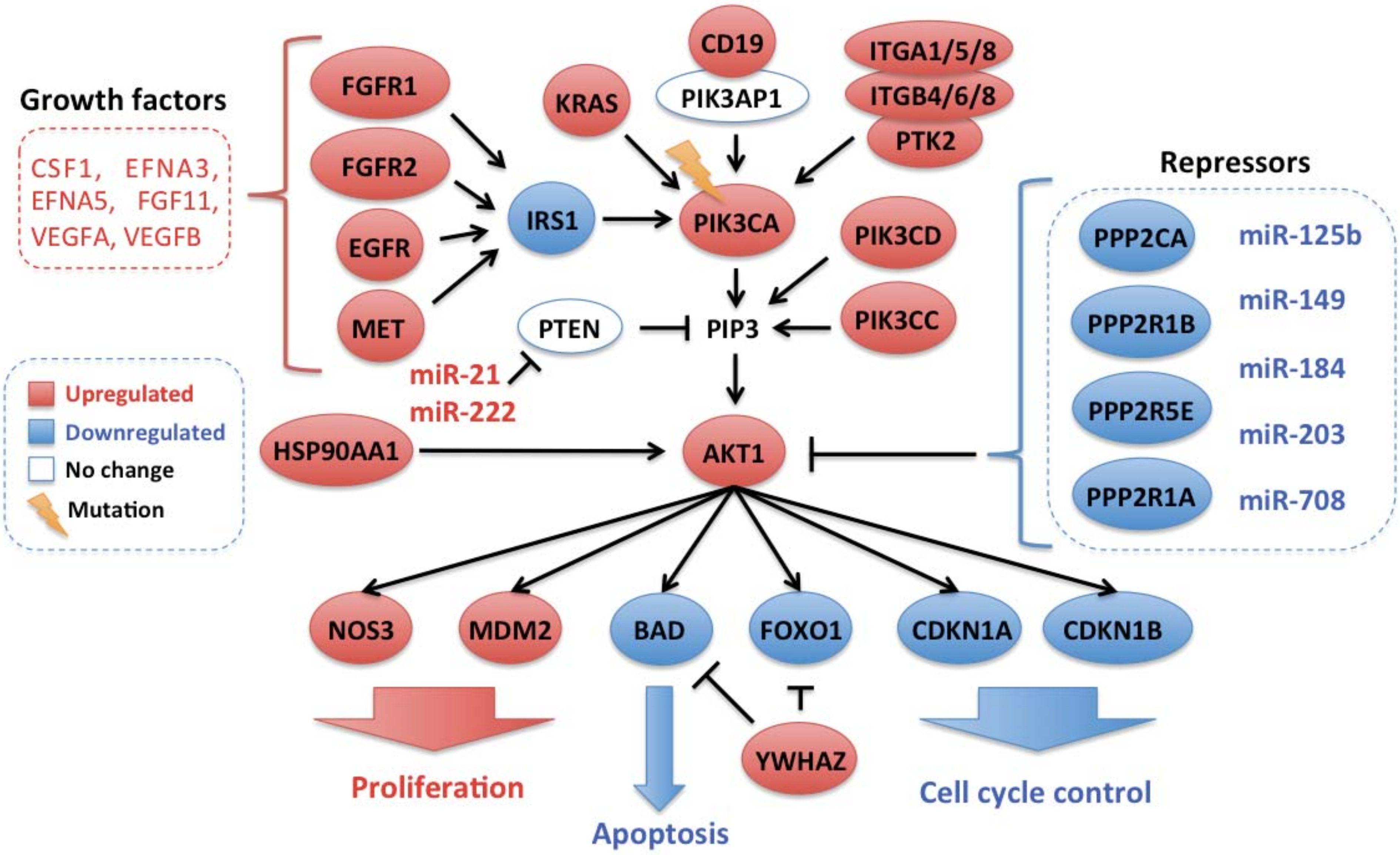

Supplement: S3 Fig — Genes with pathogenic somatic mutations and differentially expressed genes and miRNAs that regulate PI3K pathway are shown. Gene, miRNA and Pathway expression levels are depicted in red for up-regulation and blue for down-regulation; genes without differential expression are marked in white. Mutations are shown as lightning icons. (PDF) [file pone.0126762.s003.pdf]
