# Supplementary material for: Revealing the Molecular Portrait of Triple Negative Breast Tumors in an Understudied Population through Omics Analysis of Formalin-Fixed and Paraffin-Embedded Tissues
Source: PLoS One. 2015 May 11;10(5):e0126762. doi: 10.1371/journal.pone.0126762 (PMC4427337; doi:10.1371/journal.pone.0126762)

S4 Figure. Molecular alterations in FOXM1 pathway. Differentially expressed genes and miRNAs that regulate FOXM1 pathway are shown. Gene, miRNA and pathway expression levels are depicted in red for up-regulation and blue for downregulation while genes without differential expression are marked in white.

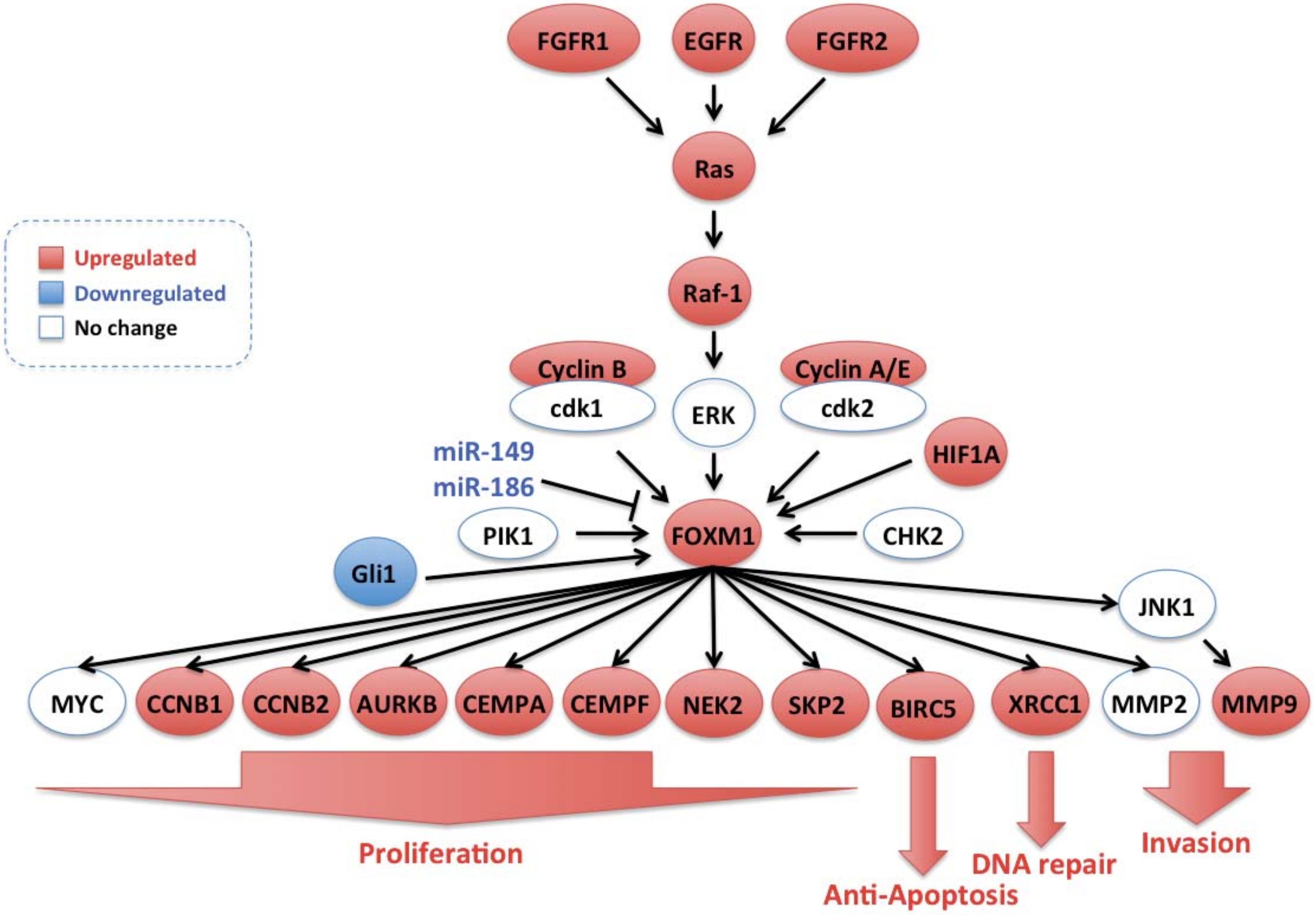

Supplement: S4 Fig — Differentially expressed genes and miRNAs that regulate FOXM1 pathway are shown. Gene, miRNA and Pathway expression levels are depicted in red for up-regulation and blue for down-regulation; genes without differential expression are marked in white. (PDF) [file pone.0126762.s004.pdf]
